# Can watching the World Cup make you mentally healthier? Positive associations between involvement in physical activities and flourishing were mediated by psychological need satisfaction

**DOI:** 10.1186/s40359-024-01861-z

**Published:** 2024-06-26

**Authors:** Hui Zhou, Bryant Pui Hung Hui, Hong Mian Yang, Anise M. S. Wu

**Affiliations:** 1 Department of Psychology, Faculty of Social Sciences, University of Macau, Macao, China; 2 Centre for Cognitive and Brain Sciences, Institute of Collaborative Innovation, University of Macau, Macao, China; 3 Department of Applied Social Sciences, Faculty of Health and Social Sciences, The Hong Kong Polytechnic University, Hong Kong, China

**Keywords:** World Cup, Physical activity, Psychological need satisfaction, Flourishing, Mental health promotion, Spectator

## Abstract

**Background:**

The current understanding of the relationship between physical activity involvement and flourishing, as well as the underlying psychological mechanisms, remains limited. Building upon the basic psychological need theory, this study investigates whether and how engaging in physical activities, either as a spectator (e.g., in sports events such as the World Cup) or a participant, explains individual differences in flourishing.

**Methods:**

A total of 1201 Hong Kong-based Chinese adults (*M*_*age*_ = 42.90, *SD* = 11.40; 54% female) completed an anonymous online survey from the quarter-final to final stages of the 2022 FIFA World Cup in December 2022.

**Results:**

Findings showed that spectator (World Cup) involvement and participant involvement were positively correlated. Moreover, both types of involvements were correlated with flourishing. In addition to the hypothesized positive association with relatedness satisfaction, spectator (World Cup) involvement was also demonstrated significant associations with autonomy and competence satisfaction. Path analysis indicated that associations between physical activities and flourishing were partially mediated by the satisfaction of all three basic psychological needs.

**Conclusions:**

This study is the first to apply the basic psychological need theory in the context of physical activity and supports its relevance in understanding individual differences in flourishing. Different stakeholders are encouraged to recognize the benefits of both spectator and participant involvements in physical activities, which may help them develop supportive strategies (e.g., physical activities with parents, sports events for spectators and participants, and funding for media/communities) in mental health promotion.

## Introduction

Mental health refers to a state of mental wellbeing, which enables individuals to cope with life’s pressure, develop abilities, and function well [1]. Its unique contribution to human development, at both individual and community levels, is critically important [1]. Given the positive roles of physical activity involvement (e.g., physical exercise) in mental health [2], this study aims to understand individual differences in flourishing among Chinese adults by examining the antecedent roles of involvement in physical activities, drawing upon the basic psychological need theory (BPNT) [3]. Flourishing encompasses optimal functioning, which includes the fulfillment of self-competence, leading a meaningful life and fostering positive relationships [4]. As a vital and informative indicator of mental health, flourishing has garnered increasing research attention [4, 5].

### Physical activities and flourishing

Building on Humphreys and Ruseski’s definition of sports [6], we adopted a comprehensive definition of physical activities. This covered not only an individual’s own bodily movement (e.g., walking, running, and playing tennis) resulting in energy expenditure as a participant (hereafter “participant involvement”), but also engagement in others’ physical activities as a spectator (hereafter “spectator involvement”), which includes paying attention, watching, and seeking information related to the sports or events of interest. Previous research has already highlighted some benefits of spectator involvement [7]. For example, spectators of the World Cup have reported higher scores on a one-item measure of subjective wellbeing compared to those who had not involved in this global event [8]. However, no studies have explored the relationship between spectator involvement in physical activities and mental health, including flourishing.

In contrast, extensive empirical evidence has demonstrated the benefits of participant involvement, including its associations with flourishing among both adolescents and adults [9, 10]. For instance, adolescents who had experiences of participant involvement reported higher levels of flourishing compared to those who did not [9]. Thus, investigating whether spectator involvement in physical activities is associated with individuals’ mental health (i.e., flourishing in this case), in addition to the direct involvement, is essential for a comprehensive understanding of the roles of physical activities. Moreover, our findings might offer insights into developing cost-effective mental health promotion strategies, such as allocating resources to support both spectators and participant involvements in physical activities. Based on existing research, this study hypothesized positive correlations between spectator (World Cup) involvement and flourishing (Hypothesis 1), as well as between participant involvement and flourishing (Hypothesis 2).

The effect of participant involvement in physical activities on flourishing has been attributed to hormonal changes. Increased endorphin and serotonin levels, as well as decreased cortisol levels, may lead to enhanced happiness and hedonic experiences, ultimately affecting individuals’ mental health [11, 12]. However, no research has examined the potential psychological mediators of this relationship. Hence, the study also aimed to address this knowledge gap by investigating the associations between involvement in physical activities, either as spectators or participants, and individuals’ flourishing from the perspective of psychological need satisfaction [3, 13].

### The mediating role of psychological need satisfaction

Psychological need has been broadly defined as an essential and crucial resource contributing to human beings’ mental health [13], and the BPNT has been applied to understand the relationship between psychological need-relevant conditions and individuals’ mental health [3, 14]. According to the BPNT, the extent to which individuals’ psychological needs are satisfied determines their optimal functioning and wellbeing [3, 15]. The BPNT proposes three psychological needs: relatedness, autonomy, and competence [3, 16, 17]. Relatedness refers to the need to have a sense of interconnectedness with others and belonging. Autonomy represents the need to feel a sense of agency or volition, knowing that one is the origin of one’s own behaviors. Competence refers to the need to express or develop one’s abilities and experience success through various tasks. The satisfaction of these three psychological needs, reflecting positive human functioning, is crucial to individuals’ flourishing and can even enhance human flourishing [17, 18]. In the literature, positive associations between psychological need satisfaction and flourishing have been well documented [19, 20]. In view of this, the present study hypothesized positive associations between the satisfaction of these three psychological needs and flourishing (Hypothesis 3).

This study aimed to extend the BPNT to the realm of physical activity involvement. From the perspectives of both spectators and participants, it examined whether individuals’ flourishing increases with physical activity involvement through the satisfaction of psychological needs. Its findings might yield a better understanding of mental health development, such as whether the satisfaction of these three psychological needs plays an equally salient role in the relationship. For the need for relatedness, this inborn desire to connect and bond with others in a social group [21] may be satisfied through spectator involvement in sports events (e.g., the World Cup in our study). For instance, a sense of belonging can be developed directly from social activities (e.g., spending time with friends and family members to watch or discuss the World Cup) [22] or indirectly from cheering or supporting one’s favorite team [23]. Apart from this, the need for relatedness may also be satisfied through participant involvement. For example, when playing football, team members must have team spirit and gather up in a joint pursuit to win. During which, they care for and help each other to achieve the common goal, allowing them to feel connected and belonged.

With respect to autonomy and competence needs, both seem to be satisfied only by participant involvement rather than spectator involvement in physical activities. Decisions about whether to join any physical activities (e.g., playing table tennis) are made based on volition (without any outside pressure) and personal interests [24], and most people feel a sense of relaxation and delight when participating in physical activities. These self-driven actions contribute to a sense of autonomy [25]. On the other hand, the need for competence can be satisfied by outperforming others (e.g., winning at table tennis) or assigning meaning to skill/performance efforts and advancements. For example, individuals can gain a sense of competence and achievement from participating in physical activities and demonstrating their physical, cognitive, and even social abilities [24].

Although empirical evidence supports the positive associations between participant involvement in physical activities and need satisfaction [26, 27], previous studies have typically incorporated a narrow definition of physical activity engagement, without considering other forms of involvement (e.g., as a spectator). Based on the BPNT [3, 14], we adopted a broader definition of physical activity involvement and hypothesized that spectator involvement (e.g., in the World Cup) would be positively correlated with relatedness satisfaction (Hypothesis 4), while participant involvement would be positively correlated with the satisfaction of all three types of psychological needs (Hypothesis 5).

**Fig. 1:**
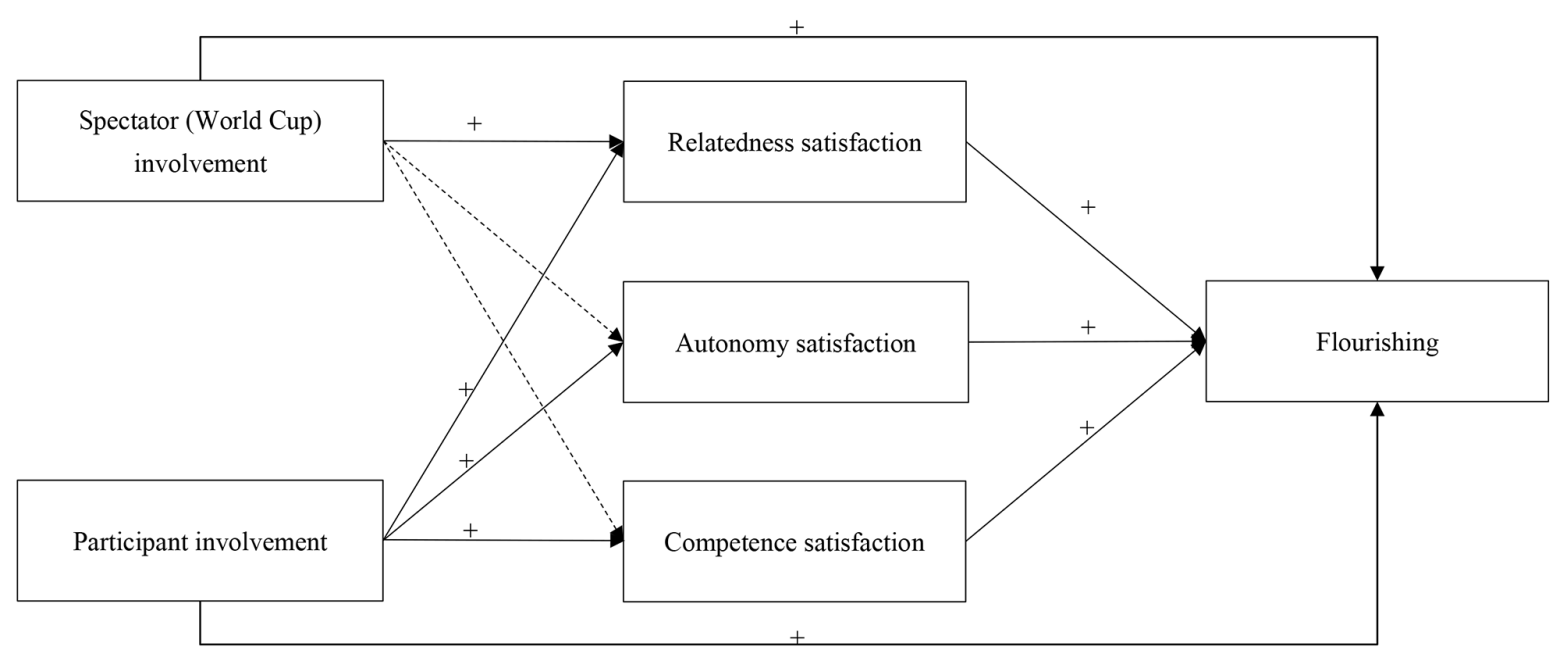
The hypothesized path model

The BPNT posits that when these basic psychological needs are satisfied, for example through involvement in physical activities as spectators or participants, beneficial effects emerge, resulting in enhanced psychological wellbeing [25, 28]. Thus, we hypothesized that relatedness satisfaction would act as a bridge between spectator (World Cup) involvement and flourishing (Hypothesis 6), while the satisfaction of all three psychological needs would mediate the associations between participant involvement and flourishing (Hypothesis 7). The conceptual model encompassing all hypothesized paths of this study is depicted in Fig. 1.

## Method

### Participants and procedure

A stratified sample of Hong Kong-based Chinese adults, balanced by age and gender, was recruited to participate in an online survey through Kantar, an international crowdsourcing platform. Data collection occurred between the 9th and 22nd of December 2022, covering the quarter-final, semi-final, and final stages of the FIFA World Cup Qatar 2022. All participants provided informed consent prior to completing a self-administered questionnaire and were made aware of their right to withdraw from the study at any time without repercussions. Ethical approval for this study was granted by the Department of Psychology at the corresponding author’s affiliated university (reference number: DPSY2022-30). A total of 1201 valid participants (*M*_*age*_ = 42.90, *SD* = 11.40; 54% female) who met the inclusion criteria (i.e., aged 18 to 64, Chinese, living in Hong Kong, and correctly answered four directed attention-check questions) were included in the present study. The age and gender distributions of the final sample had no significant deviations from the 2021 Hong Kong Population Census (age: χ^2^ (2) = 0.01, *p* = .10; gender: χ^2^ (1) = 1.95, *p* = .16) [29].

### Measures

#### Spectator (World Cup) involvement

In accordance with a previous study [30], three items were adapted to measure spectator (World Cup) involvement: “You are very interested in the World Cup,” “Your involvement in watching the World Cup is high,” and “It is very important for you to search and read information about the World Cup.” These items were rated on a 7-point Likert scale, ranging from 1 (*not at all)* to 7 (*extremely)*. A higher total score indicated a higher level of spectator (World Cup) involvement. In this study, the internal consistency of the scale was high, with a Cronbach’s α of 0.96.

#### Participant involvement

The 3-item Physical Activity Rating Scale [31] was used to measure participant involvement in physical activities. In accordance with previous studies [32, 33], participants were asked to rate items on a 5-point scale regarding the intensity (i.e., 1 = *light-intensity physical activity*, 2 = *light-to moderate-intensity physical activities*, 3 = *moderate-to vigorous-intensity physical activities*, 4 = *vigorous but not lasting physical activities with sweating a lot*, 5 = *vigorous and lasting physical activities with sweating a lot*), duration (i.e., 1 = *less than 10 min*, 2 = *10–20 min*, 3 = *21–30 min*, 4 = *31–59 min*, 5 = *60 min or above*), and frequency of their physical activities (i.e., 1 = *less than 1 time/month*, 2 = *2 to 3 times/month*, 3 = *1 to 2 times/week*, 4 = *3 to 5 times/week*, 5 = *every day*). The overall score for physical activities was calculated using the following equation: score of physical activity intensity * (score of physical activity duration – 1) * score of physical activity frequency. A higher score represented a higher level of participant involvement. In the present study, these 3 items were significantly correlated (*r* = 0.09 to 0.47, *p* < .01).

#### Psychological need satisfaction

Three subscales of the Basic Psychological Need Satisfaction and Frustration Scale [34] have been validated across China and three other countries to assess participants’ relatedness satisfaction (4 items; e.g., “I experience a warm feeling with the people I spent time with”), autonomy satisfaction (4 items; e.g., “I feel I have been doing what really interests me”), and competence satisfaction (4 items; e.g., “I feel I can successfully complete difficult tasks”). Participants rated items on a 5-point Likert scale, ranging from 1 (*strongly disagree)* to 5 (*strongly agree)*. A higher total score indicated a higher level of psychological need satisfaction. In this study, the Cronbach’s α of both relatedness satisfaction and autonomy satisfaction were 0.80, and that of competence satisfaction was 0.84, indicating adequate reliability.

#### Flourishing

The Chinese version [35] of the 8-item short form of the Flourishing Scale [4] was used to measure flourishing. The participants responded to each item (e.g., “I lead a purposeful and meaningful life”) on a 7-point Likert scale, ranging from 1 (*strongly disagree)* to 7 (*strongly agree)*. A higher total score indicated a higher level of flourishing. The Cronbach’s α of this scale was 0.93 in the present study.

#### Demographics

Participants reported their gender (1 = *male*, 2 = *female*, 3 = *other*) and age (years). Only one participant chose 3 = *other*, which was too small to be considered as a category; thus, we treated this participant’s gender as a missing value in the present study. Therefore, gender was a dichotomous variable in the data analysis.

### Data analysis

Descriptive and correlation analyses were performed using SPSS 26.0. Subsequently, the path model was tested using the lavaan package in R, employing the full information maximum likelihood estimation method with robust standard errors to address missing and nonnormal data [36, 37]. To evaluate the goodness of fit of the hypothesized path model, we followed the recommendation of Schreiber et al. [38], considering the chi-square test (χ^2^; *p* > .05), comparative fit index (CFI; ≥ 0.95), root mean square error of approximation (RMSEA; < 0.06), standardized root mean square residual (SRMR; ≤ 0.08), and Tucker-Lewis index (TLI; ≥ 0.95). For mediation testing, the total and indirect effects of variables were estimated with 95% confidential intervals based on the bias-corrected percentile method with 5,000 bootstrap samples. Statistical significance was set at *p* < .05 for all analyses.

## Results

### Bivariate correlations

Descriptive statistics and correlation coefficients of the psychological and demographic variables in this study are presented in Table 1. In line with Hypothesis 1 and Hypothesis 2, spectator (World Cup) involvement and participant involvement were significantly and positively correlated with flourishing (*r* = 0.23 and 0.18, respectively, *p* < .001). Hypothesis 3 was also substantiated by the strong positive associations between flourishing and psychological need satisfaction (*r* = 0.61 to 0.69, *p* < .001). Hypothesis 4 was supported by the positive correlation between spectator (World Cup) involvement and relatedness satisfaction (*r* = 0.17, *p* < .001). Interestingly, spectator (World Cup) involvement was also positively correlated with autonomy satisfaction and competence satisfaction (*r* = 0.19 and 0.16, respectively, *p* < .001). Lastly, Hypothesis 5 was confirmed with participant involvement demonstrating significant and positive correlations with the satisfaction of all three types of psychological needs (*r* = 0.14 to 0.16, *p* < .001).

**Table 1:** Descriptive statistics and correlations between variables (*N* = 1201)

	M	SD	Possible range	Skewness (Kurtosis)	1	2	3	4	5	6	7
1. Spectator (World Cup) involvement	13.08	5.35	3–21	–0.45 (–0.75)	1						
2. Participant involvement	14.87	16.22	0-100	1.85 (4.24)	0.15^***^	1					
3. Relatedness satisfaction	14.63	2.39	4–20	–0.82 (1.78)	0.17^***^	0.14^***^	1				
4. Autonomy satisfaction	14.44	2.48	4–20	–0.83 (1.27)	0.19^***^	0.16^***^	0.56^***^	1			
5. Competence satisfaction	14.20	2.54	4–20	–0.82 (1.19)	0.16^***^	0.14^***^	0.65^***^	0.54^***^	1		
6. Flourishing	37.02	8.33	8–56	–0.53 (0.36)	0.23^***^	0.18^***^	0.63^***^	0.61^***^	0.69^***^	1	
7. Age	42.90	11.40	18–64	–0.04 (–1.08)	–0.12^***^	–0.04	0.17^***^	0.08^**^	0.14^***^	0.13^***^	1
8. Gender (1 = male, 2 = female)	--	--	--	-- (--)	–0.24^***^	–0.10^**^	–0.05	–0.01	–0.04	–0.02	–0.07^*^

Note: ^*^p < .05, ^**^p < .01, ^***^p < .001

### Path analysis

**Fig. 2:**
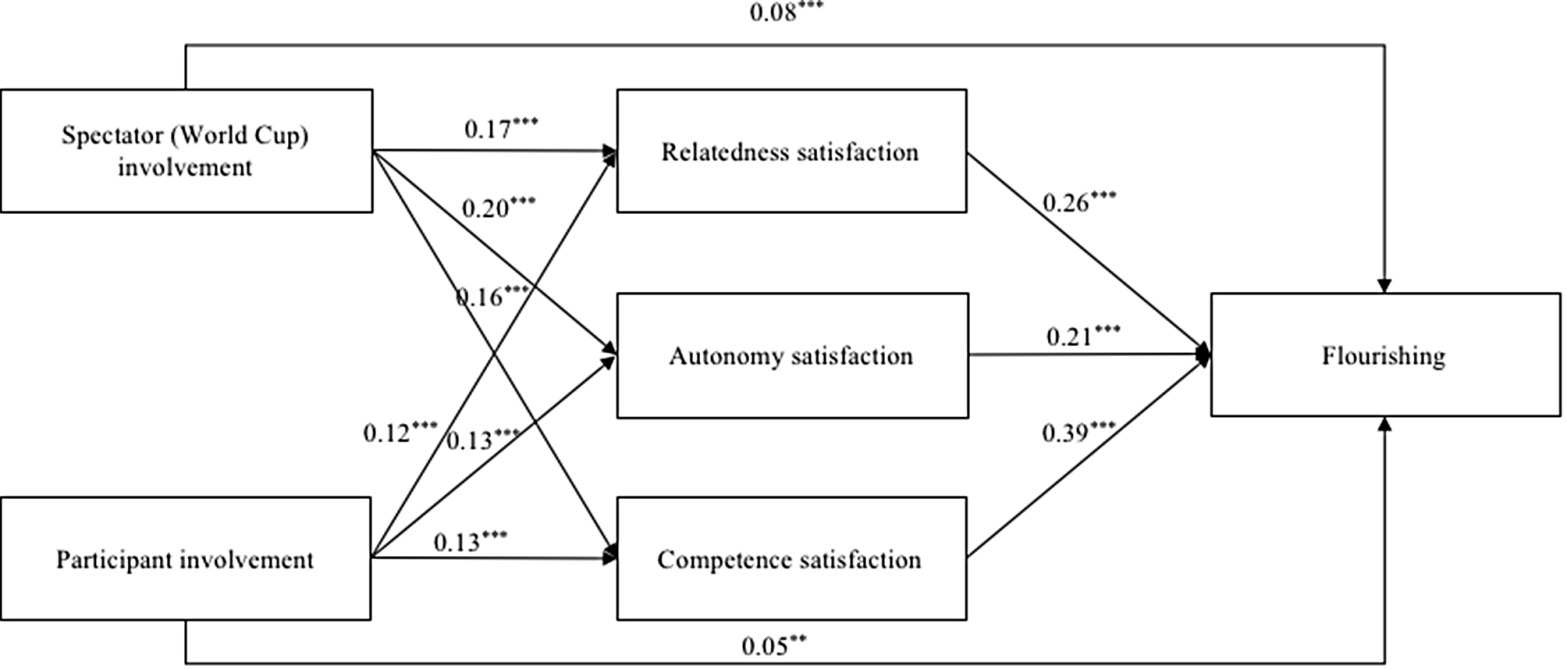
The final path model with standardized estimates. Note: ^**^p < .01, ^***^p < .001. This model has controlled for the effects of age and gender on the correlated variables. Moreover, the covariances of spectator (World Cup) and participant involvement as well as the error covariances of autonomy satisfaction, relatedness satisfaction, and competence satisfaction were allowed and statistically significant (*p* < .001)

Controlling for the effects of age and gender on their correlated variables, the conceptual path model was tested with path analysis. Its model fit was poor, χ^2^ (8) = 649.17, *p* < .001, CFI = 0.57, RMSEA = 0.26, 90% CI [0.25, 0.27], SRMR = 0.16, TLI = –0.50. In accordance with modification indices, residual covariances between the three dimensions of psychological need satisfaction (i.e., relatedness satisfaction, autonomy satisfaction, competence satisfaction) were allowed to improve the model fit. The modified path model showed a good fit with the data, χ^2^ (5) = 8.85, *p* = .36, CFI = 1.00, RMSEA = 0.03, 90% CI [0.00, 0.05], SRMR = 0.02, TLI = 0.99. As shown in Fig. 2, the standardized coefficients of all hypothesized paths were statistically significant.

As shown in Table 2, the total effect of involvement in physical activities (i.e., spectator [World Cup] involvement and participant involvement) on flourishing was found to be significant, with a value of 0.39 (95% CI [0.32, 0.45]). In addition to the mediating effect of relatedness satisfaction (*β* = 0.04, 95% CI [0.02, 0.06]) as proposed by Hypothesis 6, the indirect effects from spectator (World Cup) involvement to flourishing via autonomy satisfaction (*β* = 0.04, 95% CI [0.03, 0.06]) and competence satisfaction (*β* = 0.06, 95% CI [0.04, 0.09]) were also significant. Furthermore, the association between participant involvement and flourishing was significantly mediated by autonomy satisfaction (*β* = 0.03, 95% CI [0.02, 0.04]), relatedness satisfaction (*β* = 0.03, 95% CI [0.02, 0.05]), and competence satisfaction (*β* = 0.05, 95% CI [0.03, 0.07]), which was in line with Hypothesis 7.

**Table 2:** Testing the pathways of the mediation model

Path	β	95% CI (lower, upper)	Statistical significance
Total effect of spectator (World Cup) involvement and participant involvement on flourishing	0.39	(0.32, 0.45)	Significant
Total effect of spectator (World Cup) involvement on flourishing	0.23	(0.17, 0.29)	Significant
Indirect effect of spectator (World Cup) involvement on flourishing via relatedness satisfaction	0.04	(0.02, 0.06)	Significant
Indirect effect of spectator (World Cup) involvement on flourishing via autonomy satisfaction	0.04	(0.03, 0.06)	Significant
Indirect effect of spectator (World Cup) involvement on flourishing via competence satisfaction	0.06	(0.04, 0.09)	Significant
Total effect of participant involvement on flourishing	0.16	(0.11, 0.21)	Significant
Indirect effect of participant involvement on flourishing via relatedness satisfaction	0.03	(0.02, 0.05)	Significant
Indirect effect of participant involvement on flourishing via autonomy satisfaction	0.03	(0.02, 0.04)	Significant
Indirect effect of participant involvement on flourishing via competence satisfaction	0.05	(0.03, 0.07)	Significant

## Discussion

In light of the crucial role of mental health in individual and societal development [1], this study aimed to expand the application of the BPNT [3] to understand the relationship between flourishing and the involvement in physical activities as spectators and participants. Our pioneering attempt was successful in revealing their positive correlations, which were partially mediated by the satisfaction of all three psychological needs.

This study addressed the research gap regarding the connection between flourishing and the involvement in physical activities in its broader definition. It found significant and positive total and direct effects of involvement as spectators or participants, which were positively correlated, on individuals’ flourishing. Previous studies [9, 10] have already revealed the positive role of participant involvement. However, the role of spectator involvement has only been examined and reported in one previous study, where World Cup spectators reported a higher score on a single-item measure of subjective wellbeing than non-spectators [8]. Hence, the current findings not only corroborate the positive role of spectator involvement but also show that such association remains significant even after controlling for participant involvement in sports and physical activities.

Furthermore, the study illustrated that the BPNT was effective in explaining the associations between physical activities and flourishing, as the satisfaction of all three psychological needs played a partial mediating role in this relationship. Our findings on the positive associations between participant involvement and relatedness satisfaction were consistent with a previous study, which found that participant involvement in physical activities was correlated with relatedness satisfaction among Chinese older adults [26]. In addition, our study provided empirical evidence for the positive associations of participant involvement with autonomy and competence satisfaction.

Coinciding with our hypothesis, spectator involvement during the World Cup was positively correlated with relatedness satisfaction. Besides, it had a positive association with autonomy and competence satisfaction. This suggested that spectator involvement is a self-driven behavior similar to participant involvement, and individuals can decide whether to engage based on their own volition and self-endorsed interests. Making decisions on which games to watch, which teams to support, and how to support them may help to develop a sense of autonomy [39]. Additionally, spectators’ identification [40] and shared success with their teams give rise to a sense of competence. In particular, when their teams win, they may perceive it as an affirmation of their ability to pick the capable teams. Thus, self-driven behaviors and team identification contribute to the satisfaction of autonomy and competence, respectively.

Our findings suggest that both spectator (World Cup) involvement and participant involvement contributed to the satisfaction of needs for autonomy, relatedness, and competence, all of which were associated with high levels of flourishing. Similar associations have been demonstrated among Chinese young gamers and adult gamblers in previous studies [19, 20]. These findings align with the BPNT, which posits that psychological need-related factors (e.g., physical activities in our study) fulfill humans’ basic and innate needs [3, 14]. When these needs are satisfied, individuals will have more resources for optimal functioning and self-actualization [3, 17, 18]. The theory emphasizes that humans have fundamental and positive psychological propensities [41] that can be revealed not only through internal fulfillment but also under supportive physical conditions. Such conditions may include a favorable context for people to participate in sports and physical activities (whether personal games or global events like the World Cup) across various means and platforms (i.e., offline vs. online).

Within the theoretical framework of the BPNT, our findings demonstrated that involvement in physical activities, as both spectators and participants, explained individual differences in flourishing, which is a crucial indicator of mental health [5]. Our study suggested that effort and resources for mental health promotion should be allocated to encourage both spectator and participant involvements in physical activities. In terms of spectator involvement, parents can watch global sports events (e.g., the World Cup) live or on television with their children as family activities. This can promote family interactions and cohesion, leading to better wellbeing among youths [42, 43]. Other stakeholders can make contributions as well [44]. For example, more sports events or friendly matches can be organized and held by schools, so that students can watch the competitions and support professional athletes or their peers. Policymakers should also ensure adequate funding for communities to offer more accessible spaces for spectators to enjoy these activities, including listening/viewing media for sport and physical activity events [6]. Thus, new media and technology (e.g., podcasts and social media platforms) should be invested in and advocated to facilitate the interactions between event organizers, athletes, and spectators. For individuals unable to participate in physical activities due to physical limitations (e.g., injuries or illness), engaging as spectators in events where others are physically active may improve their flourishing in less physically demanding ways. Notably, an increase in sports events presents potential risks, such as the rise of sports gambling [45]. Thus, policymakers must carefully put forward harm-reduction strategies, such as tighter regulations for gambling advertising during sports games and events to discourage irresponsible gambling [46].

On the other hand, participant involvement in physical activities should also be promoted, given its salient role in mental health [2]. Compared to previous studies on Chinese individuals [32, 47], the present study reported a relatively low score for participant involvement in physical activities (i.e., *M* = 14.87 versus 22.08 and 28.66 using the same measure). Excluding the impact of anti-pandemic policies, the current data suggest that effective measures are needed to promote participant involvement in physical activities among Hong Kong residents. Taking into account the positive relation between parental support and participant involvement in physical activities among teenagers [48], parents are suggested to enroll their children in sports clubs of their interests, co-participate in physical activities, and encourage their children to use school/community resources. In addition to incorporating more physical activities into the daily curriculum, schools can consider partnering with non-profit organizations to offer after-school/summer camps packed with physical activities (e.g., outdoor adventures). For policymakers, they should emphasize the importance of participant involvement while allocating more funding to design and build a supportive environment. In particular, they can set up more sports facilities (e.g., free bicycle parking sites and sports courts) to enhance public participation in physical activities [49].

Based on the BPNT [3, 14], we proposed and tested a conceptual model with directional paths, in which mental health (i.e., flourishing) was the outcome variable, physical activity involvements as spectators and/or participants were the potential antecedents, and the three psychological needs were the mediating variables. This model is theoretically driven but other alternative models do exist. For example, it is plausible that people with higher levels of flourishing tend to engage in more physical activities. However, the cross-sectional design of the present study did not allow the empirical examination of causal or even temporal relationships among physical activities, psychological need satisfaction, and flourishing. Therefore, the replicability of our findings should be tested with longitudinal and experimental designs for further evaluation of the current and alternative conceptual models of the variables.

Furthermore, our findings should be interpreted with caution due to other limitations. First, the study only recruited Chinese adults living in Hong Kong through online means, which may impact its generalizability to other ethnic or age groups. Hence, further research is needed to test the applicability of our findings to different populations. Second, data regarding physical activities were self-reported, making them susceptible to inaccuracies owing to memory lapses. Future studies should consider incorporating objective measurement data of physical activities, such as electronic data records of participant involvement in physical activities and engagement tracking of sports events. Finally, our findings revealed that psychological need satisfaction partially mediated the link between involvement in various types of physical activities and flourishing. Future studies should explore other possible psychological mechanisms (e.g., mitigating negative emotions) [50] to further understand these relationships.

## Conclusion

The present study established the positive associations between physical activities and individuals’ flourishing from the perspective of both spectators and participants. Our research is the first to apply the BPNT to understand the prominent roles of physical activity involvement in flourishing, revealing that the satisfaction of all three basic psychological needs mediates this relationship. The findings not only address the limitations of previous studies, which have mainly focused on individuals’ own involvement in physical activities, but also offer empirical evidence for a theory-based, psychological mechanism underlying positive associations between the involvement in physical activities and mental health promotion. Furthermore, our study explains what parents, schools, and policymakers can do to facilitate and encourage spectator and participant involvements in physical activities, which may serve as an effective approach to improving mental health.

## Data Availability

The dataset generated and/or analyzed during the current study will be available from the corresponding author on reasonable request.
